# 3-Carb­oxy-2-methoxy­phenyl­boronic acid

**DOI:** 10.1107/S1600536808029504

**Published:** 2008-09-20

**Authors:** Sergiusz Luliński

**Affiliations:** a Warsaw University of Technology, Faculty of Chemistry, Noakowskiego 3, 00-664 Warsaw, Poland

## Abstract

The mol­ecular structure of the title compound, 3-COOH-2-CH_3_O—C_6_H_3_B(OH)_2_ or C_8_H_9_BO_5_, is stabilized in part due to the presence of an intra­molecular O—H⋯O hydrogen bond. In the crystal structure, mol­ecules are linked by inter­molecular O—H⋯O hydrogen bonds, generating a two-dimensional sheet structure aligned parallel to the (11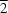) plane.

## Related literature

For structures of other carboxy­phenyl­boronic acids, see: SeethaLekshmi & Pedireddi (2007 ▶); Soundararajan *et al.* (1993 ▶). For the application of carboxy­phenyl­boronic acids in crystal engineering, see: (Aakeröy *et al.*, 2005 ▶; SeethaLekshmi & Pedireddi, 2006 ▶). For structural characterization of related *ortho*-alk­oxy aryl­boronic acids, see: Dabrowski *et al.* (2006 ▶); Dąbrowski *et al.* (2008 ▶); Yang *et al.* (2005 ▶). For the synthesis of the title compound, see: (Kurach *et al.*, 2008 ▶).
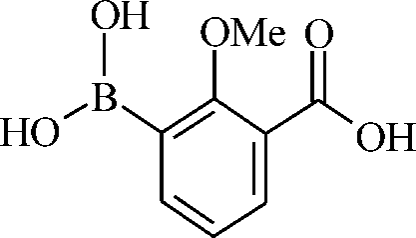


## Experimental

### 

#### Crystal data

C_8_H_9_BO_5_
*M* *_r_* = 195.96
Triclinic, 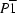

*a* = 4.8451 (5) Å
*b* = 7.7564 (7) Å
*c* = 12.1064 (9) Å
α = 79.476 (7)°
β = 79.575 (7)°
γ = 76.125 (8)°
*V* = 429.75 (7) Å^3^
*Z* = 2
Mo *K*α radiation
μ = 0.12 mm^−1^
*T* = 100 (2) K
0.32 × 0.20 × 0.14 mm

#### Data collection

Kuma KM4 CCD diffractometer
Absorption correction: multi-scan (*CrysAlis RED*; Oxford Diffraction 2005 ▶) *T*_min_ = 0.95, *T*_max_ = 0.98
12229 measured reflections
2106 independent reflections
1526 reflections with *I* > 2σ(*I*)
*R*_int_ = 0.018

#### Refinement

*R*[*F* ^2^ > 2σ(*F* ^2^)] = 0.035
*wR*(*F* ^2^) = 0.101
*S* = 1.05
2106 reflections
163 parameters
All H-atom parameters refined
Δρ_max_ = 0.35 e Å^−3^
Δρ_min_ = −0.26 e Å^−3^

### 

Data collection: *CrysAlis CCD* (Oxford Diffraction (2005 ▶); cell refinement: *CrysAlis RED* (Oxford Diffraction (2005 ▶); data reduction: *CrysAlis RED*; program(s) used to solve structure: *SHELXS97* (Sheldrick, 2008 ▶); program(s) used to refine structure: *SHELXL97* (Sheldrick, 2008 ▶); molecular graphics: *DIAMOND* (Brandenburg, 1999 ▶); software used to prepare material for publication: *SHELXTL* (Sheldrick, 2008 ▶).

## Supplementary Material

Crystal structure: contains datablocks I. DOI: 10.1107/S1600536808029504/tk2303sup1.cif

Structure factors: contains datablocks I. DOI: 10.1107/S1600536808029504/tk2303Isup2.hkl

Additional supplementary materials:  crystallographic information; 3D view; checkCIF report

## Figures and Tables

**Table:** 

B1—O2	1.3443 (15)
B1—O3	1.3461 (16)
B1—C9	1.5661 (17)
C6—O8	1.2607 (13)
C6—O7	1.3044 (14)

**Table:** 

O2—B1—C9—C14	18.65 (16)
C5—O4—C10—C9	93.35 (12)
O8—C6—C11—C10	177.57 (10)

**Table 2:** Hydrogen-bond geometry (Å, °)

*D*—H⋯*A*	*D*—H	H⋯*A*	*D*⋯*A*	*D*—H⋯*A*
O2—H2⋯O3^i^	0.802 (19)	1.96 (2)	2.7572 (13)	172.6 (18)
O3—H3⋯O4	0.84 (2)	2.06 (2)	2.7283 (12)	136.8 (19)
O3—H3⋯O2^ii^	0.84 (2)	2.45 (2)	3.0538 (14)	130.2 (19)
O7—H8⋯O8^iii^	1.03 (2)	1.60 (2)	2.6255 (11)	177.0 (16)

Symmetry codes: (i) 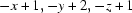; (ii) 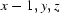; (iii) 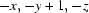.

**Fig. 1.**
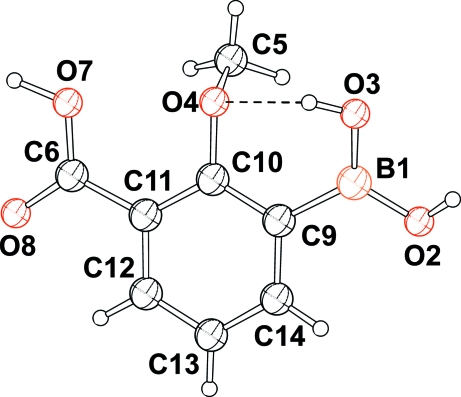
The molecular structure of (I) showing the atom-labelling scheme. The intramolecular hydrogen bond is shown as a dashed lines. Displacement ellipsoids for all non-H atoms are drawn at the 50% probability level.

**Fig. 2.**
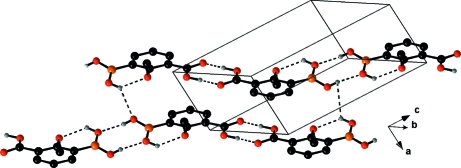
The hydrogen-bonding pattern for (I). Hydrogen bonds are shown as dashed lines.

## supplementary crystallographic information

### Comment

The presence of a carboxyl group in a molecule of arylboronic acid provides an increased potential for extended supramolecular organization (SeethaLekshmi & Pedireddi, 2007). The promising properties of carboxyphenylboronic acids in crystal engineering (Aakeröy *et al.*, 2005; SeethaLekshmi & Pedireddi, 2006) prompted us to determine the structure of the title compound, (I).

The molecular structure of (I) shows the boronic groups possesses an *exo*-*endo* conformation and is slightly twisted with respect to the benzene ring (Table 1). The methoxy group is twisted almost perpendicularly with respect to the aromatic ring. The *endo*-oriented OH group is engaged in an intramolecular O—H···O hydrogen bonds with the methoxy O atom, resulting in the formation of a six-membered ring. This motif is generally typical of structures of *ortho*-alkoxyarylboronic acids (Yang *et al.*, 2005; Dąbrowski *et al.*, 2006). The carboxyl group is almost coplanar with respect to the benzene ring. The molecules are linked *via* almost linear O—H···O bridges in a "head-to-head, tail-to-tail" fashion, *i.e.*, equivalent groups interact with each other forming two alternate centrosymmetric dimeric motifs, Table 2. As a result, an infinite, zigzag chain is formed (Fig. 2). The chain structure resembles the situation found for the related 2-methoxy-1,3-phenylenediboronic acid (Dąbrowski *et al.*, 2008), where single molecules are linked *via* homomeric (SeethaLekshmi & Pedireddi, 2007) hydrogen-bonding interactions of non-equivalent boronic groups.

The 1D supramolecular architecture extends through cross-linking weak O—H···O bonds between twisted boronic groups. As a result a 2D array is formed, aligned parallel to the (11–2) plane. In conclusion, the intermolecular hydrogen-bonding interactions of boronic and carboxyl groups result in the formation of the infinite chain structure. Chains are interconnected by means of weaker hydrogen-bonds, thus forming the layer structure.

### Experimental

The compund was prepared according to the published procedure (Kurach *et al.*, 2008). Crystals suitable for single-crystal X-ray diffraction analysis were grown by slow evaporation of a solution of the acid (0.15 g) in ethyl acetate/acetone (10 ml, 1:1).

### Refinement

All hydrogen atoms were located in difference syntheses and refined freely so that O-H = 0.802 (19) - 1.03 (2) Å and C-H = 0.942 (17) - 1.029 (17) Å.

### Crystal data

**Table:**

C_8_H_9_BO_5_	*V* = 429.75 (7) Å^3^
*M**_r_* = 195.96	*Z* = 2
Triclinic, *P*1	*F*(000) = 204
Hall symbol: -P 1	*D*_x_ = 1.514 Mg m^−^^3^
*a* = 4.8451 (5) Å	Melting point: 429-432 K K
*b* = 7.7564 (7) Å	Mo *K*α radiation, λ = 0.71073 Å
*c* = 12.1064 (9) Å	µ = 0.12 mm^−^^1^
α = 79.476 (7)°	*T* = 100 K
β = 79.575 (7)°	Prismatic, colourless
γ = 76.125 (8)°	0.32 × 0.20 × 0.14 mm

### Data collection

**Table:**

Kuma KM4 CCD diffractometer	2106 independent reflections
Radiation source: fine-focus sealed tube	1526 reflections with *I* > 2σ(*I*)
graphite	*R*_int_ = 0.018
Detector resolution: 8.6479 pixels mm^-1^	θ_max_ = 28.6°, θ_min_ = 3.0°
ω scan	*h* = −6→6
Absorption correction: multi-scan (CrysAlis RED; Oxford Diffraction 2005)	*k* = −10→10
*T*_min_ = 0.95, *T*_max_ = 0.98	*l* = −16→16
12229 measured reflections	

### Refinement

**Table:**

Refinement on *F*^2^	Primary atom site location: structure-invariant direct methods
Least-squares matrix: full	Secondary atom site location: difference Fourier map
*R*[*F*^2^ > 2σ(*F*^2^)] = 0.035	Hydrogen site location: inferred from neighbouring sites
*wR*(*F*^2^) = 0.101	All H-atom parameters refined
*S* = 1.05	*w* = 1/[σ^2^(*F*_o_^2^) + (0.0643*P*)^2^] where *P* = (*F*_o_^2^ + 2*F*_c_^2^)/3
2106 reflections	(Δ/σ)_max_ = 0.001
163 parameters	Δρ_max_ = 0.35 e Å^−^^3^
0 restraints	Δρ_min_ = −0.26 e Å^−^^3^

### Special details

**Table:**

Geometry. All e.s.d.'s (except the e.s.d. in the dihedral angle between two l.s. planes) are estimated using the full covariance matrix. The cell e.s.d.'s are taken into account individually in the estimation of e.s.d.'s in distances, angles and torsion angles; correlations between e.s.d.'s in cell parameters are only used when they are defined by crystal symmetry. An approximate (isotropic) treatment of cell e.s.d.'s is used for estimating e.s.d.'s involving l.s. planes.
Refinement. Refinement of *F*^2^ against ALL reflections. The weighted *R*-factor *wR* and goodness of fit *S* are based on *F*^2^, conventional *R*-factors *R* are based on *F*, with *F* set to zero for negative *F*^2^. The threshold expression of *F*^2^ > σ(*F*^2^) is used only for calculating *R*-factors(gt) *etc*. and is not relevant to the choice of reflections for refinement. *R*-factors based on *F*^2^ are statistically about twice as large as those based on *F*, and *R*- factors based on ALL data will be even larger.

### Fractional atomic coordinates and isotropic or equivalent isotropic displacement parameters (Å^2^)

**Table:**

	*x*	*y*	*z*	*U*_iso_*/*U*_eq_	
B1	0.5522 (3)	0.79514 (18)	0.40716 (11)	0.0168 (3)	
O2	0.77114 (19)	0.78932 (13)	0.46290 (7)	0.0247 (2)	
O3	0.3223 (2)	0.93263 (13)	0.41255 (8)	0.0295 (3)	
O4	0.20761 (17)	0.81061 (11)	0.23177 (7)	0.0181 (2)	
C5	0.3211 (3)	0.92945 (19)	0.13926 (12)	0.0300 (3)	
C6	0.2362 (2)	0.50663 (16)	0.11119 (9)	0.0165 (3)	
O7	0.04150 (18)	0.65037 (11)	0.08598 (7)	0.0199 (2)	
O8	0.27658 (18)	0.36943 (11)	0.06341 (7)	0.0216 (2)	
C9	0.5714 (2)	0.63677 (16)	0.34035 (9)	0.0168 (3)	
C10	0.3987 (2)	0.65025 (15)	0.25686 (9)	0.0147 (3)	
C11	0.4152 (2)	0.50426 (15)	0.20035 (9)	0.0162 (3)	
C12	0.6083 (3)	0.34441 (17)	0.22948 (10)	0.0200 (3)	
C13	0.7810 (3)	0.32923 (17)	0.31153 (11)	0.0232 (3)	
C14	0.7620 (3)	0.47419 (17)	0.36588 (10)	0.0204 (3)	
H2	0.729 (4)	0.869 (2)	0.5008 (16)	0.052 (5)*	
H3	0.204 (5)	0.903 (3)	0.3808 (19)	0.083 (7)*	
H5A	0.506 (4)	0.956 (2)	0.1564 (13)	0.043 (4)*	
H5B	0.187 (3)	1.040 (2)	0.1310 (13)	0.043 (4)*	
H5C	0.361 (3)	0.8735 (19)	0.0712 (13)	0.031 (4)*	
H8	−0.080 (4)	0.638 (2)	0.0276 (16)	0.067 (6)*	
H12	0.612 (3)	0.245 (2)	0.1950 (12)	0.025 (4)*	
H13	0.906 (3)	0.217 (2)	0.3314 (12)	0.030 (4)*	
H14	0.878 (3)	0.4602 (18)	0.4239 (11)	0.024 (4)*	

### Atomic displacement parameters (Å^2^)

**Table:**

	*U*^11^	*U*^22^	*U*^33^	*U*^12^	*U*^13^	*U*^23^
B1	0.0178 (7)	0.0218 (7)	0.0127 (6)	−0.0063 (5)	−0.0035 (5)	−0.0036 (5)
O2	0.0229 (5)	0.0308 (5)	0.0255 (5)	−0.0031 (4)	−0.0095 (4)	−0.0147 (4)
O3	0.0305 (5)	0.0277 (5)	0.0376 (6)	0.0014 (4)	−0.0205 (4)	−0.0182 (4)
O4	0.0218 (4)	0.0159 (4)	0.0178 (4)	−0.0018 (3)	−0.0072 (3)	−0.0040 (3)
C5	0.0408 (8)	0.0200 (7)	0.0276 (7)	−0.0065 (6)	−0.0072 (6)	0.0031 (6)
C6	0.0174 (6)	0.0191 (6)	0.0147 (6)	−0.0076 (5)	−0.0012 (4)	−0.0034 (5)
O7	0.0212 (4)	0.0209 (5)	0.0201 (4)	−0.0022 (4)	−0.0103 (3)	−0.0048 (3)
O8	0.0280 (5)	0.0219 (5)	0.0193 (4)	−0.0088 (4)	−0.0060 (4)	−0.0069 (4)
C9	0.0156 (6)	0.0220 (6)	0.0139 (6)	−0.0063 (5)	−0.0025 (4)	−0.0023 (5)
C10	0.0146 (5)	0.0156 (6)	0.0142 (5)	−0.0042 (4)	−0.0012 (4)	−0.0024 (4)
C11	0.0167 (6)	0.0179 (6)	0.0149 (6)	−0.0062 (5)	−0.0007 (4)	−0.0028 (5)
C12	0.0215 (6)	0.0169 (6)	0.0222 (6)	−0.0051 (5)	−0.0012 (5)	−0.0045 (5)
C13	0.0210 (6)	0.0197 (6)	0.0257 (7)	−0.0013 (5)	−0.0036 (5)	0.0007 (5)
C14	0.0178 (6)	0.0273 (7)	0.0164 (6)	−0.0054 (5)	−0.0049 (5)	−0.0002 (5)

### Geometric parameters (Å, °)

**Table:**

B1—O2	1.3443 (15)	C6—C11	1.4971 (16)
B1—O3	1.3461 (16)	O7—H8	1.03 (2)
B1—C9	1.5661 (17)	C9—C14	1.3933 (17)
O2—H2	0.802 (19)	C9—C10	1.3993 (16)
O3—H3	0.84 (2)	C10—C11	1.4059 (16)
O4—C10	1.3813 (14)	C11—C12	1.3931 (17)
O4—C5	1.4317 (15)	C12—C13	1.3825 (18)
C5—H5A	1.029 (17)	C12—H12	0.938 (15)
C5—H5B	0.942 (17)	C13—C14	1.3799 (18)
C5—H5C	0.968 (15)	C13—H13	0.955 (15)
C6—O8	1.2607 (13)	C14—H14	0.951 (14)
C6—O7	1.3044 (14)		
			
O2—B1—O3	119.64 (11)	C14—C9—B1	119.41 (10)
O2—B1—C9	118.18 (11)	C10—C9—B1	122.49 (10)
O3—B1—C9	122.16 (10)	O4—C10—C9	118.38 (10)
B1—O2—H2	109.5 (13)	O4—C10—C11	120.37 (10)
B1—O3—H3	104.3 (15)	C9—C10—C11	121.25 (11)
C10—O4—C5	113.51 (10)	C12—C11—C10	118.37 (10)
O4—C5—H5A	110.1 (9)	C12—C11—C6	116.96 (10)
O4—C5—H5B	109.1 (10)	C10—C11—C6	124.66 (10)
H5A—C5—H5B	107.3 (13)	C13—C12—C11	121.05 (11)
O4—C5—H5C	108.6 (9)	C13—C12—H12	120.4 (9)
H5A—C5—H5C	110.3 (13)	C11—C12—H12	118.5 (9)
H5B—C5—H5C	111.4 (13)	C14—C13—C12	119.70 (12)
O8—C6—O7	122.02 (10)	C14—C13—H13	121.3 (9)
O8—C6—C11	119.10 (10)	C12—C13—H13	118.9 (9)
O7—C6—C11	118.88 (10)	C13—C14—C9	121.55 (11)
C6—O7—H8	114.0 (10)	C13—C14—H14	118.6 (8)
C14—C9—C10	118.08 (11)	C9—C14—H14	119.8 (8)
			
O2—B1—C9—C14	18.65 (16)	O4—C10—C11—C6	0.00 (17)
O3—B1—C9—C14	−159.62 (11)	C9—C10—C11—C6	179.25 (10)
O2—B1—C9—C10	−162.83 (11)	O8—C6—C11—C12	−3.26 (16)
O3—B1—C9—C10	18.89 (18)	O7—C6—C11—C12	176.22 (10)
C5—O4—C10—C9	93.35 (12)	O8—C6—C11—C10	177.57 (10)
C5—O4—C10—C11	−87.38 (13)	O7—C6—C11—C10	−2.95 (16)
C14—C9—C10—O4	179.50 (10)	C10—C11—C12—C13	−0.30 (18)
B1—C9—C10—O4	0.96 (16)	C6—C11—C12—C13	−179.52 (11)
C14—C9—C10—C11	0.24 (17)	C11—C12—C13—C14	0.18 (19)
B1—C9—C10—C11	−178.30 (10)	C12—C13—C14—C9	0.16 (19)
O4—C10—C11—C12	−179.16 (10)	C10—C9—C14—C13	−0.37 (17)
C9—C10—C11—C12	0.09 (17)	B1—C9—C14—C13	178.22 (10)

### Hydrogen-bond geometry (Å, °)

**Table:**

*D*—H···*A*	*D*—H	H···*A*	*D*···*A*	*D*—H···*A*
O2—H2···O3^i^	0.802 (19)	1.96 (2)	2.7572 (13)	172.6 (18)
O3—H3···O4	0.84 (2)	2.06 (2)	2.7283 (12)	136.8 (19)
O3—H3···O2^ii^	0.84 (2)	2.45 (2)	3.0538 (14)	130.2 (19)
O7—H8···O8^iii^	1.03 (2)	1.60 (2)	2.6255 (11)	177.0 (16)

Symmetry codes: (i) −*x*+1, −*y*+2, −*z*+1; (ii) *x*−1, *y*, *z*; (iii) −*x*, −*y*+1, −*z*.
